# Comparison of erector spinae plane block and rhomboid intercostal block for postoperative pain management in patients undergoing unilateral breast surgery

**DOI:** 10.1186/s12871-026-03655-9

**Published:** 2026-03-06

**Authors:** Gülnihal Avcı, Sevim Cesur Okan, Hadi Ufuk Yörükoğlu, Can Aksu, Alparslan Kuş

**Affiliations:** 1 Department of Anesthesiology and Reanimation, Kocaeli University, Kocaeli, Turkey; 2 Department of Anesthesiology and Reanimation, Ordu Korgan State Hospital, Tepe Mahalle Sağlık Ocağı Sokak No:30 , Korgan, Ordu Türkiye

**Keywords:** Rhomboid intercostal block (RIB), Erector spinae plane block (ESPB), Postoperative analgesia, Multimodal analgesia, Breast surgery

## Abstract

**Purpose:**

The incidence of moderate-to-severe postoperative pain following breast surgery is approximately 50%. Effective postoperative analgesia is essential, as uncontrolled pain may increase morbidity, impair recovery, and reduce quality of life. This study aimed to compare the efficacy of the erector spinae plane block (ESPB) and rhomboid intercostal block (RIB) for postoperative pain management, based on 24-hour morphine consumption. Secondary outcomes included intraoperative opioid use, numerical rating scale (NRS) scores at rest and during movement, time to first analgesic request, and complication rates.

**Methods:**

Women > 18 years of age, American Society of Anesthesiologists (ASA) physical status I–III, undergoing elective unilateral breast surgery were enrolled. Exclusion criteria included lack of cooperation, chronic analgesic use, drug hypersensitivity, infection at the puncture site, spinal or paravertebral deformity, and coagulation disorders. Patients were randomized into ESPB (*n* = 42) or RIB (*n* = 42) groups using a computer-generated sequence. Thirty minutes before surgery, 20 mL 0.25% bupivacaine was administered under ultrasound guidance.

**Results:**

Data from 84 patients were analyzed. Twenty-four–hour morphine consumption did not differ significantly between groups (*p* > 0.05). NRS scores at 1, 3, 6, 12, and 24 h were also comparable (*p* > 0.05). Hemodynamic parameters and intraoperative opioid requirements showed no significant differences. Postoperative nausea and vomiting occurred in one ESPB and three RIB patients (*p* > 0.05). No block-related complications were observed.

**Conclusion:**

Within a standardized multimodal analgesia regimen for unilateral breast surgery, ESPB and RIB yielded comparable postoperative opioid consumption and pain scores. Clinicians may therefore select either technique according to their own expertise and institutional practice.

## Introduction

Postoperative pain is a significant phenomenon that increases morbidity and mortality, lowers quality of life, and affects patients at least as much as surgery and medical treatment [1]. Inadequate postoperative pain management can adversely impact multiple systems [2]. Due to the complex innervation of the breast, numerous regional techniques have been described for pain control, but the search for the ideal technique continues. The anterior and lateral cutaneous branches of the second, third, and sixth intercostal nerves, the supraclavicular nerve from C3-C4, cutaneous branches from the cervical plexus, the pectoral nerve, thoracodorsal nerve, and long thoracic nerve all contribute to postoperative pain following breast surgery [2, 3]. Retraction of the pectoralis major muscle and fascia leads to myofascial pain, and drain placement exacerbates postoperative pain [4]. Current postoperative pain management favors multimodal analgesia, incorporating both opioid and non-opioid regional techniques.

Fascial plane blocks are increasingly utilized as components of multimodal postoperative analgesia, with novel techniques continually described. Various effect mechanisms of fascial plane blocks have been determined as a result of cadaver and imaging studies. These blocks exhibit their effects by distributing local anesthetic to the interstitial compartments of the injected plane. In addition, local anesthetic can also be disseminated to neighboring muscles or tissues via bulk flow through pores in fascial tissue and/or diffusion, or local anesthetic can exhibit a systemic effect via vascular absorption [5]. Erector spinae plane block (ESPB) was described by Forero et al. [6] in 2016, and is currently employed for anesthesia and postoperative analgesia in numerous types of surgery, including oncological and non-oncological breast surgery, and thoracic, abdominal, and lumbar region surgeries [7–11]. ESPB involves ultrasound (USG)-guided injection of local anesthetic to the fascial area between the vertebral transverse processes and erector spinae muscle at the determined vertebral level. The local anesthetic spreads cranially and caudally in this potential space for three to six levels, depending on the direction of the block needle. Medial and lateral spread of local anesthetic is also possible, although this is limited by the ribs and the thoracolumbar fascia surrounding the erector spinae muscle [6].

Rhomboid intercostal block (RIB), also described by Elsharkawy et al. [12] in 2016, is a regional anesthesia technique of proven efficacy in the management of postoperative pain following thoracotomy, lung transplantation, and radical mastectomy, and also in the treatment of myofascial pains [13–15]. It is applied to the region known as the triangle of auscultation, located along the lower medial border of the scapula. It is bounded by the trapezius superiorly, the latissimus dorsi inferiorly, and the vertebral margins of the scapula laterally. The floor of the triangle consists of the rhomboid major (inferior), the lateral part of the erector spinae muscle, and the serratus anterior muscle. The floor lies over the sixth and seventh ribs and their interna and external intercostal muscles. The RIB has been reported to provide effective analgesia in both the anterior and posterior hemithoraces affecting the T2-T9 intercostal nerves in the caudocephalic area as a result of the injection of local anesthetic over the ribs and intercostal muscles beneath the rhomboid major muscle at the T5-T6 levels [12].

This study evaluated ESPB versus RIB for postoperative pain in breast surgery patients, primarily assessing 24-hour morphine consumption, and secondarily intraoperative opioid use, numerical rating scale (NRS) scores, time to first analgesic, and complication rates.

## Methods

### Study design

This study was designed as a prospective, randomized, single-blind trial. Patients and the postoperative pain assessor were blinded to group allocation, whereas the anesthesiologists performing the blocks were not, due to the nature of the intervention. Ethical approval was obtained from the Kocaeli University Medical Faculty Clinical Research Ethics Committee (KAEK/08.bI.02), and the study was supported by the Scientific Research Projects Coordination Unit of Kocaeli University (Project number: 3316). Approval from Clinical Trials was obtained while the study was ongoing (NCT06177665). The reporting of this randomized controlled trial adheres to the CONSORT 2025 guidelines. The study was conducted in the Kocaeli University Medical Faculty Research Hospital (Türkiye) operating room following receipt of written informed consent from the patients in accordance with the ethical principles concerning medical research involving humans in the World Medical Association Declaration of Helsinki.

American Society of Anesthesiologists (ASA) physical status I-III women aged over 18 undergoing elective unilateral breast conserving surgery (BCS) or modified radical mastectomy (MRM) (with/without axially lymph node dissection) were included in the study. Non-cooperative patients, those with histories of chronic pain-related analgesia use, with sensitivity to the drugs to be employed, with infection in the procedural area, with spinal/paravertebral deformity, or with coagulation disorder were excluded.

### Block procedure

The patients were assigned to one of two groups, one undergoing ESPB (the ESPB group; *n* = 42) and the other RIB (the RIB group; *n* = 42) using computer-supported software (https://www.randomizer.org).

All patients were taken to the block area in the operating room half an hour before surgery and were again informed about the procedure to be carried out. Following routine ASA monitoring (electrocardiogram (EKG), SpO2 (oxygen saturation), and noninvasive blood pressure (NIBP) monitoring, oxygenation was provided with a 2 L/min nasal cannula, and premedication was performed with 0.03 mg/kg intravenous (iv) midazolam. Once the side marking had been checked, the patients were placed in the prone position and ultrasound-guided block applications were performed by SC, HUY, and GA.

**Fig. 1:**
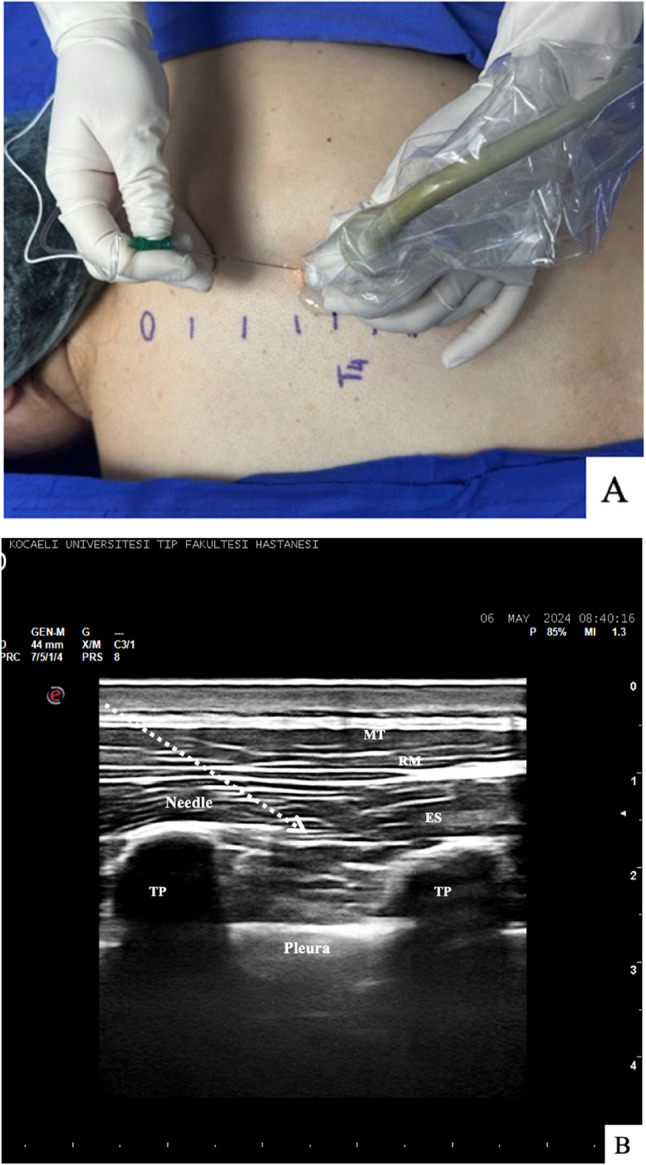
Erector Spinae Plane Block Procedure. (**A**) and its Ultrasonographic Appearance (**B**) MT: Muscles Treapezius RM: Muscles Rhomboid Major ES: Muscles Erector Spinae TP: Transverse Process

In the ESPB group, the spinous processes were visualized with the instalment on the side to be operated of a high-frequency linear ultrasound probe (MyLab 6, Esaote, Florence, Italy) in the midline sagittal plane. The T4 spinous process was identified by counting from the C7 vertebra and the T7 vertebra at the lower end of the scapula, which were adopted as landmarks. After initial estimation using surface landmarks, the final vertebral level and transverse processes were confirmed based on sonoanatomy before advancing the needle. The ultrasound probe was installed 2–3 cm laterally to the T4 spinous process. Following the determination of the transvers process, the trapezius, rhomboid major, and erector spinae muscles were identified. A 22-gauge 5–8 cm block needle with an extension line (B. Braun, Melsungen, Germany) visible on ultrasound was advanced in a craniocaudal direction through the skin, subcutaneous tissue, and trapezius, rhomboid major, and erector spinae muscles toward the transverse process using an in-plane approach. Once the needle tip had arrived beneath the erector spinae muscle, 0.5–1 mL physiological saline solution was administered, and the location of the needle was confirmed by observing the spread of the solution under the erector spinae muscle. Twenty milliliters of 0.25% bupivacaine was then applied to this area (Fig. 1).

**Fig. 2:**
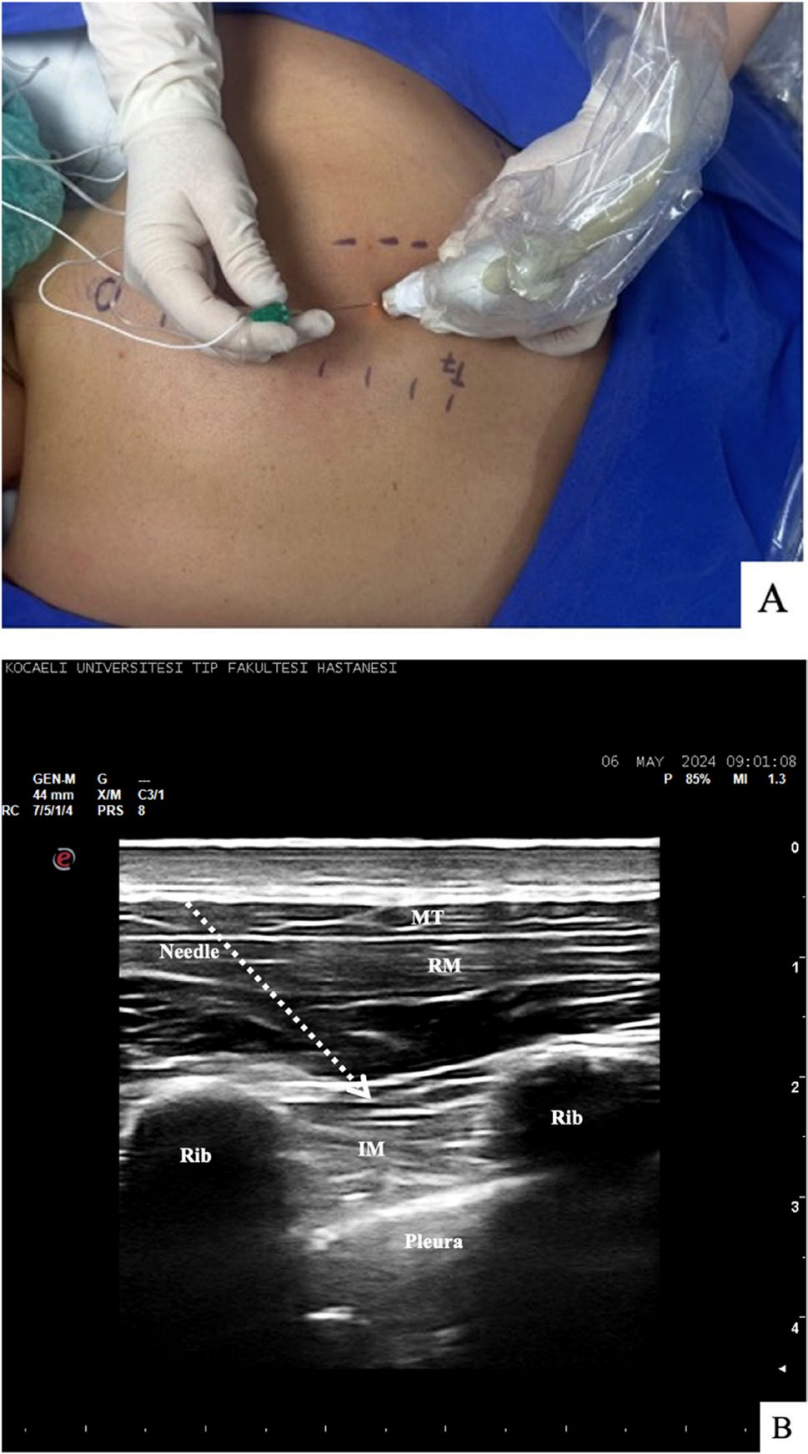
Rhomboid Intercostal Block Procedure. (**A**) and its Ultrasonographic Appearance (**B**) M.T: Muscles Treapezius R.M: Muscles Rhomboid Major I.M: Intercostal muscles

In the RIB group a high-frequency linear ultrasound was placed on the medial margin of the scapula in the transverse plane at the T5-T6 level on the side to be operated. The trapezius, rhomboid major, and intercostal muscles, the pleura and the lung were visualized. The ultrasound probe was then rotated 90 degrees, and the skin, subcutaneous tissue, and trapezius and rhomboid major muscles were passed using a 22 gauge 5–8 cm needle visible at USG with an in-plane approach in the sagittal plane. When the tip of the needle was beneath the rhomboid major muscle, its location was confirmed with the administration of 0.5–1.5 mL physiological saline solution. Twenty milliliters of 0.25% bupivacaine was then applied to this area (Fig. 2).

### Intraoperative and postoperative management

Following standard ASA monitoring (EKG, SpO2, and NIBP), anesthesia was induced with 2–3 mg/kg propofol, and airway safety was established using an appropriately sized laryngeal mask airway (LMA; ProSeal, Teleflex, USA). Maintenance anesthesia was established using remifentanil infusion with a 2% sevoflurane O2/air (1/2) mixture. The depth of anesthesia was established by keeping Bi-Spectral Index (BIS) monitoring between 40 and 60. The remifentanil infusion dosage was adjusted in accordance with hemodynamic changes (with appropriate titration being provided when heart rate and blood pressure fell below or exceeded 20% of the baseline values). At the end of surgery, the LMA was removed when a sufficient respiration rate and depth were achieved, and the patient was taken to the postoperative recovery room for observation. Patients with modified Aldrete scores above 9 were transferred to the ward.

Thirty minutes before the end of surgery, the patients in both groups also received 1 gram paracetamol, 100 mg tramadol, and 8 mg ondansetron. All patients’ analgesic requirements were met using a patient-controlled analgesia (PCA) device delivering morphine (Acute Pain Manager, Abbott Laboratories, North Chicago, IL, USA). The PCA device was set to release morphine at a concentration of 0.5 mg/mL in a volume of 100 mL, a 2 mL bolus, a locking time of 8 min, and six compressions an hour. Pain was evaluated in line with the training provided for the patients during preoperative visits using a numeric rating scale (NRS) (with 0 indicating no pain and 10 the most severe pain). Patients’ NRS scores, morphine consumption and demand levels, additional analgesia demand and amounts, and nausea and vomiting complaints were recorded at postoperative hours 1, 3, 6, 12, and 24 by a pain technician blinded to the study groups. Patients with NRS scores exceeding 4 were given 20 mg tenoxicam for postoperative analgesia, and a 3 mg bolus was administered if NRS scores remained above 4 after 30 min.

### Outcome measures

The primary outcome of this study was the evaluation of total morphine consumption at postoperative 24 h. The secondary outcome measures were intraoperative analgesia consumption, NRS values at rest and while coughing at postoperative hours 1, 3, 6, 12, and 24, total morphine consumption at 1, 3, 6, 12 and 24 h, and times of first analgesia requirements. Patients’ demographic data, perioperative heart rate, blood pressure, Spo2, and BIS values were recorded in the perioperative period. Postoperative nausea/vomiting and block-related complications were also noted.

### Statistical analysis

A preliminary study was conducted in our clinic involving 30 patients, with 15 patients each in the RIB and ESPB groups. Based on this pilot data, the mean 24-hour postoperative morphine consumption was 2.93 ± 0.9 mg in the RIB group and 4.02 ± 1.0 mg in the ESPB group. For the primary outcome (24-h postoperative morphine consumption), the trial was designed as a non-inferiority study, testing whether RIB was not clinically worse than ESPB. The non-inferiority margin (Δ) was prespecified as 0.545 mg, based on pilot data from our institution and clinical judgement in the context of a multimodal analgesic regimen. The primary analysis compared the mean 24-h morphine consumption between groups (RIB – ESPB) and calculated the corresponding two-sided 90% confidence interval (equivalent to a one-sided α = 0.05). The required sample size was calculated using the following formula for non-inferiority trials: n= [2(Z_1−α_ +Z_1−β_)^2^⋅σ ^2^]/(Δ) ^2^ where σ is the pooled standard deviation, Δ is the non-inferiority margin, Z_1−α_ is the critical value for the significance level (α = 0.05), and Z_1−β_ is the critical value corresponding to a power of 80%. Using this formula, and assuming a pooled standard deviation of approximately 0.95 (derived from pilot data), a total of 38 patients per group was required to detect non-inferiority with 80% power and a one-sided α of 0.05. To account for a possible dropout rate of 10%, we enrolled a total of 84 patients in the study.

Statistical analyses were performed on IBM SPSS version 20.0 software (IBM Corp., Armonk, NY, USA). Normality of distribution was analyzed using the Kolmogorov-Smirnov and Shapiro-Wilk tests. Normally distributed variables were expressed as mean ± standard deviation, and non-normally distributed variables as median (25th-75th percentile) values. Categorical variables were expressed as frequency (percentage) values. Intergroup comparisons were performed using the t test and the Mann-Whitney U test. Relationships between categorical variables were determined using chi square analysis. Dependent group comparisons for numerical variables were conducted using Friedman two-way ANOVA. Dunn’s test was applied for multiple comparisons. Dependent group comparisons for categorical variables were performed using Cochran’s Q test. A p value < 0.05 as regarded as significant in hypothesis tests. Although 24-h morphine consumption showed a modest right-skewed distribution, a t test-based confidence interval for the mean difference was used for the non-inferiority analysis, as is commonly recommended for continuous outcomes in non-inferiority trials with moderate sample sizes; medians and interquartile ranges are additionally reported for descriptive purposes.

## Results

**Fig. 3:**
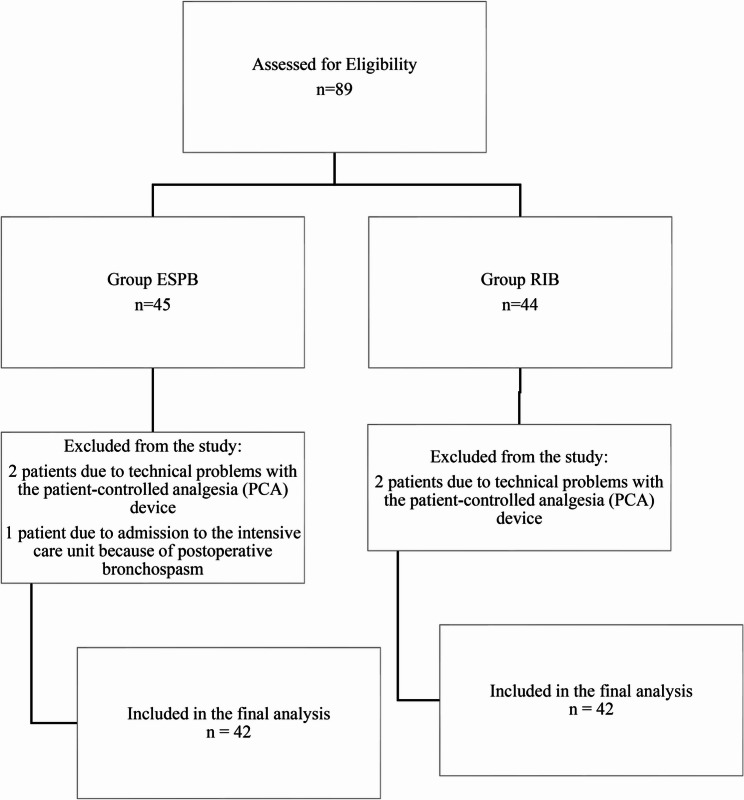
CONSORT flow diagram of the study. Group ESPB: Patients who received erector spinae plane block; Group RIB: Patients who received rhomboid intercostal block PCA: Patient-controlled analgesia

Between July 2023 and June 2024, eighty-nine patients undergoing unilateral breast surgery were included in the study. Forty-five patients were initially included in the ESPB group, but two were excluded due to technical problems with the PCA device and one due to transfer to intensive care as a result of postoperative bronchospasm. Forty-four patients were originally included in the RIB group, but two were subsequently excluded due to technical difficulties with the PCA device. The data for 84 of the 89 patients were thus included in the analysis (Fig. 3).

**Fig. 4:**
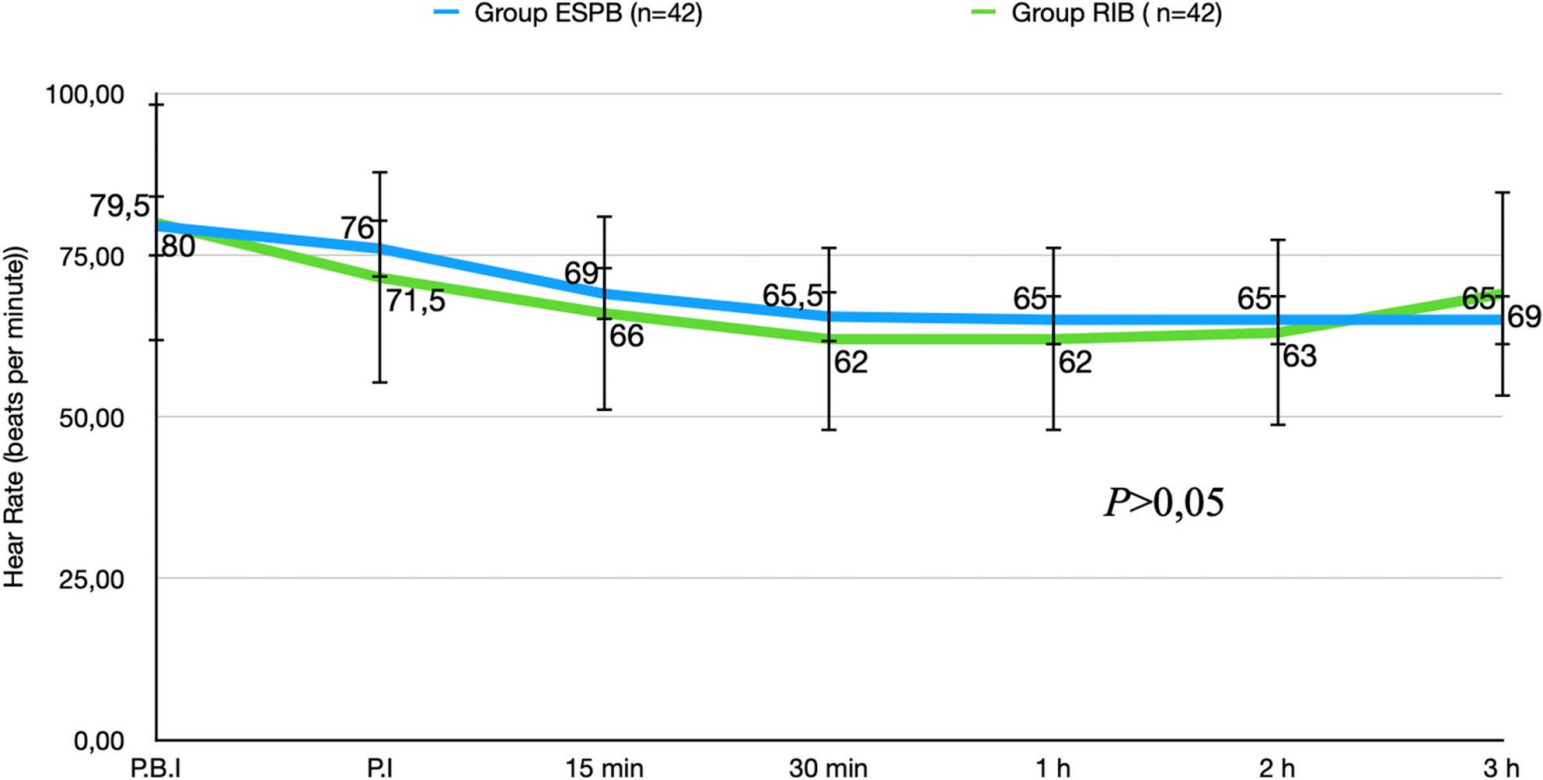
Intraoperative heart rate data. *P<0.05 was considered statistically significant, Group ESPB: Patients who received erector spinae plane block; Group RIB: Patients who received rhomboid intercostal block; P.I.: Post-induction, P.B.I.: Pre-induction, MAP: Mean arterial pressure

**Fig. 5:**
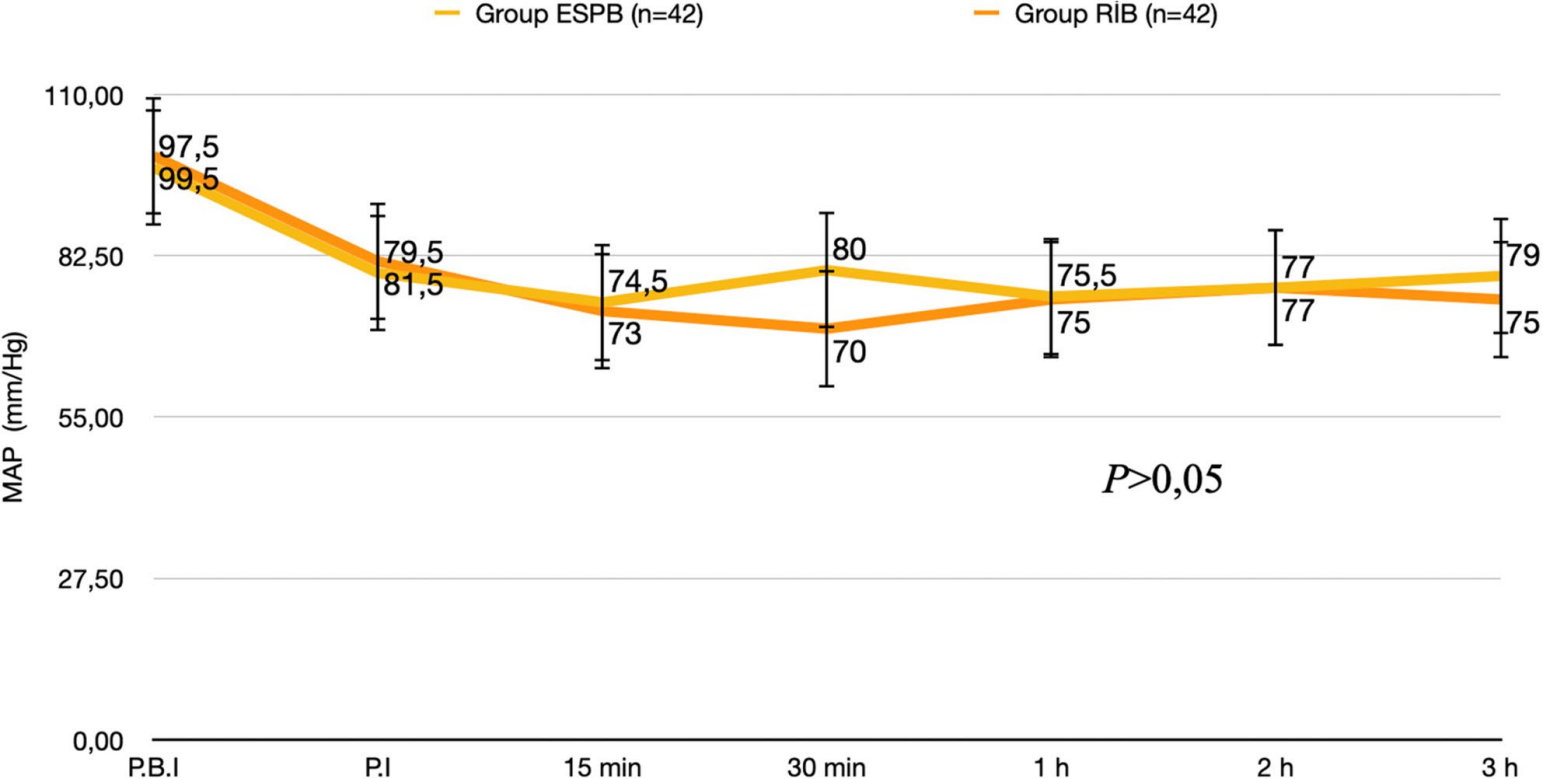
Intraoperative MAP (mmHg) data. *P<0.05 was considered statistically significant, Group ESPB: Patients who received erector spinae plane block; Group RIB: Patients who received rhomboid intercostal block; P.I.: Post-induction, P.B.I.: Pre-induction, MAP: Mean arterial pressure

No significant differences were observed between the groups in terms of the patients’ demographic data, ASA physical status, or accompanying diseases (*P* > 0.05) (Table 1). No significant intergroup differences were also determined in terms of operative time or the nature of the surgery (*P* > 0.05) (Table 2). Intraoperative hemodynamic data were also similar between the two groups (*P* > 0.05). (Fig. 4) (Fig. 5).

**Table 1:** Anthropometric and clinical characteristics of patients in ESPB and RIB groups

Variable	ESPB Group (*n* = 42)	Group RİB (*n* = 42)	*P* value
Age (years)	53.67 ± 11.765	51.64 ± 13.577	0.467
BMI (kg/m^2^)	28.1010 ± 5.02614	28.6240 ± 5.49774	0.650
ASA Physical Status (I/II/III), n (%)	4(%9.5)/34(%81.0)/4(%9.5)	12(%28.6)/28(%66.7)/2(%4.8)	0.067
Systemic Disease (1/2/3/4), n	13/12/9/3	4/8/4/7	0.273

Values are presented as mean ± standard deviation or as number of patients and percentages

Group ESPB: Patients who received erector spinae plane block

Group RIB: Patients who received rhomboid intercostal block

*BMI* Body Mass Index

*ASA * American Society of Anesthesiologists Physical Status Classification;

Systemic Disease Categories: 1: Diabetes mellitus, 2: Hypertension, 3: Hypertension and Diabetes mellitus, 4: Hypothyroidism

**Table 2:** Surgical informations

	Group ESPB (*n* = 42)	Group RİB (*n* = 42)	*P* value
Duration of surgery (min)	120(87.50–138.75)	90(75.00–150.00)	0.570
Surgery Type BCS/mastectomy n (%)	27(%64.3)/15(%35.7)	34(%55.7)/8(%19.0)	0.142
Axillary Lymph Node Dissection (yes/no) n (%)	20(%52.4)/22(%47.6)	20(%52.4)/22(%47.6)	1.000

Values are presented as median (percentiles 25–75) or patient numbers and percent (%)

Group ESPB: Patients who received erector spinae plane block

Group RIB: Patients who received rhomboid intercostal block

*BCS *Breast Conserving Surgery

Total postoperative 24-hour morphine consumption levels, the primary study outcome, were similar between the two groups (*P* > 0.05). Mean 24-h morphine consumption was 3.19 ± 2.63 mg in the ESPB group and 2.95 ± 2.13 mg in the RIB group (RIB – ESPB mean difference − 0.24 mg; 90% CI − 1.10 to 0.62 mg; 95% CI − 1.26 to 0.79 mg). Intraoperative remifentanil consumption was also similar (*P* > 0.05) (Table 3). Additional analgesia requirements were required in both groups at the first hour, and 14 patients from the ESPB group and 16 from the RIB group were given non-steroid anti-inflammatory drugs (NSAIDs) (P:0.949). No significant differences in morphine consumption were observed between the groups at postoperative hours 1, 3, 6, 12, or 24 (*P* > 0.05) (Table 4). Twenty-four hour total morphine consumption was 2 mg (1–5 mg) in the ESPB group and 2 mg (1–4 mg) in the RIB group (*P* > 0.05) (Table 3). At postoperative follow-up, no significant differences were determined in at rest/in motion NRS values at hours 1, 3, 6, 12, and 24 in the ESPB group and hours 1, 3, 6, 12, and 24 in the RIB group (*P* > 0.05) (Table 5).

**Table 3:** Intraoperative and postoperative opioid consumption

	Group ESPB (*n* = 42)	Group RİB (*n* = 42)	*P* value
Total remifentanil consumption(mcq)	335.00 (198.00–480.00)	250(175–505)	0.840
Total morphine consumption in 24 h (mg)	2.00 (1.00–5.00)	2.00 (1.00–4.00)	0.960

Values are presented as median (25th–75th percentiles)

Group ESPB: Patients who received erector spinae plane block;

Group RIB: Patients who received rhomboid intercostal block

*mg * Milligrams

**Table 4:** Morphine Consumptions at the Postoperative 1 st, 3nd, 6th, 12th, 24th Hour

Time Point	Group ESPB (n = 42) Morphine Consumption (mg)	Group RIB (n = 42) Morphine Consumption (mg)	*P value*
1 st hour	1.00 (1.00–2.00)	1.00 (1.00-1.00)	0.690
3nd hour	2.00 (1.00–2.25)	2.00 (1.00–3.00)	0.401
6th hour	2.00 (1.00–4.00)	2.00 (1.00–3.25)	0.945
12th hour	2.00 (1.00–4.25)	2.00 (1.00–4.00)	0.842
24th hour	2.00 (1.00–5.00)	2.00 (1.00–4.00)	0.960

Group ESPB: Patients who received erector spinae plane block

Group RIB: Patients who received rhomboid intercostal block

Values are presented as median (25th–75th percentile)

**Table 5:** NRS scores at rest and during movement at the 1 st, 3rd, 6th, 12th, and 24th postoperative hours (A-B)

A. NRS Scores at Rest			
Time Point	ESPB Group (*n* = 42)	RIB Group (*n* = 42)	*P* value
1 st hour	3.00 (1.75–4.00)	3.00 (2.00–4.00)	0.875
3nd hour	2.00 (0.00–3.00)	2.00 (0.00–3.00)	0.290
6th hour	0.00 (0.00–2.00)	0.00 (0.00–2.00)	0.581
12th hour	0.00 (0.00–0.00)	0.00 (0.00–0.00)	0.514
24th hour	0.00 (0.00–0.00)	0.00 (0.00–0.00)	0.554
B. NRS Scores During Movement			
Time Point	ESPB Group (*n* = 42)	RIB Group (*n* = 42)	*P* value
1 st hour	4.00 (2.00–5.00)	4.00 (2.75–5.00)	0.458
3nd hour	2.00 (0.00–4.00)	3.00 (1.50–4.00)	0.284
6th hour	2.00 (0.00–3.00)	1.00 (0.00–2.00)	0.092
12th hour	0.00 (0.00–2.00)	0.00 (0.00–0.00)	0.182
24th hour	0.00 (0.00–0.00)	0.00 (0.00–0.00)	0.729

Values are presented as median (25–75 percentile) Group ESPB: Patients who received erector spinae plane block. Group RIB: Patients who received rhomboid intercostal block

Postoperative nausea-vomiting was observed in one patient from the ESPB group and three from the RIB group, the difference between the groups being statistically insignificant (*P* > 0.05). No complications associated with the block applications were observed.

## Discussion

This study investigated the efficacy of the ESPB and RIB on postoperative pain in patients undergoing unilateral breast surgery. Twenty-four hour total morphine consumption was similar between the two groups. No significant differences were also determined between the groups in terms of the secondary outcomes, intraoperative analgesic consumption, NRS values at rest and in movement, times to first analgesic requirements, and complication rates. 

The incidence of moderate-severe postoperative pain following breast surgery is approximately 50% [16]. Failure to achieve sufficient postoperative pain control is associated with increasing morbidity, impairment of functional status and quality of life, delayed healing times, prolonged opioid use, and increased health care costs [1]. Fascial plane blocks are becoming increasingly used as one step in multimodal analgesia in the management of postoperative pain, and novel blocks are being described on a regular basis. One of the most popular of these, the ESPB, was first described by Forero et al. [6] in 2016, following which it was employed for postoperative analgesia in various indications, and in the treatment of chronic pain [17]. One of the first publications in which the ESPB was used for postoperative analgesia in breast surgery was a study by Gürkan et al. [18], who reported that it significantly reduced postoperative morphine consumption. In that study, the ESPB was applied in the same manner as in the present research, under USG guidance with 20 ml 0.25% bupivacaine from the T4 level, with the patient in the prone position. Several studies have now proved the analgesic efficacy of the ESPB in breast surgery [19–24]. Since the ESPB has been shown to provide effective analgesia when applied at the T4 level in the majority of studies, we also applied it from that level in the current research.

The RIB was first described in 2016 by Elsharkawy et al. [12], and has been reported to provide effective analgesia in both hemithoraces, anterior and posterior. Despite being a fascial plane block described at the same time as the ESPB, the RIB has a more limited area of application [13, 25–27].

The use of the RIB for breast surgery analgesia was first published as a case report in 2018 by Tulgar et al. [28]. It was applied under USG guidance from the T5-T6 level using 20 mL 0.25% bupivacaine, 10 mL 2% lidocaine, and 10 ml saline solution with the patient in the decubitus position, with a postoperative NRS of 2–3/10. Yayık et al. [29]. applied the block from the T5-T6 level in to cases of breast reduction surgery, using 5 mL 2% lidocaine, 10 mL bupivacaine, and 5 mL saline solution, and described it as effective.

In addition to published case reports, the first randomized, controlled study involving the use of the RIB in breast surgery was performed by Altıparmak et al. [30] in 2020. That study involved 56 patients, the RIB being applied under USG guidance from the T5 level, with the patient in the lateral decubitus position after intubation, using 30 mL 0.25% bupivacaine. In contrast to the present study, the block as applied with the patient in the lateral decubitus position, under general anesthesia, and with a higher volume of local anesthetic. In the present study, the block was applied to all patients in the block area 30 min prior to surgery. We elected to perform the procedure with the patient under sedation since we thought that placing patients in position for the block application under general anesthesia would lead to a loss of time and performance in terms of cost-effective use of operating rooms. Total 24-hour postoperative morphine consumption was 5 mg (4–7 mg, median 25–75) in the RIB group and 10 mg (8–13 mg, median 25–75) in the ESPB group (*P* < 0.001), while postoperative NRS values were similar between the two groups. In terms of previous 24-hour postoperative morphine consumption in previous studies, consumption in our RIB group (2 mg, 1–4 mg, median 25–75) was lower than that reported by Altıparmak et al. The lower opioid consumption in the present study may be attributable to the use of different surgical techniques to Altıparmak et al., since we also included patients not undergoing axillary dissection. Damage to the intercostobrachial nerve plays a role in axillary dissection pain, although branches of the thoracodorsal nerve and brachial plexus are also involved in the innervation of this region [31]. A previous cadaver study reported that the RIB included the axillary area, while others have reported that the ESPB is effective against axillary pain when performed from the T4 level or higher (T2) [12, 32]. We think that the non-homogeneous nature of our patient group affected postoperative opioid consumption.

In a study comparing the RIB with the pectoserratus block (PSPB) and a control group in breast surgery, Çiftçi et al. [33]. reported that the RIB yielded superior results to a control group, while exhibiting similar efficacy to the PSPB group. Ninety patients were included in that study, 30 in each group. Both blocks were performed under USG guidance with 30 mL 0.25% bupivacaine. Postoperative fentanil consumption was similar between the two block groups, at 29.33 µg for the RIB and 30 µg for the PSPB, and lower than in the control group (92.67 µg) (*P* < 0.001). VAS values at rest and during motion were also similar in all three groups. In terms of morphine equivalence (1 mg iv morphine being equivalent to 0.066 µg iv fentanil), opioid consumption was similar to that in the RIB group in the present study (2 mg, 1–4 mg, median 25–75). Nausea-vomiting in the block groups in that study (two patients in the RIB group, five in the PSPB group, and 11 in the control group) was also similar to that in the present research (three patients in the RIB group and one in the ESPB group). We think that the use of multimodal analgesia management, low postoperative opioid consumption, and the use of ondansetron in our routine practice are all beneficial in terms of nausea-vomiting.

Kültüroğlu et al. [34] compared the postoperative analgesic efficacy of the RIB and interpectoral–pectoserratus block (IPPB) + PSPB in breast surgery in 2014 and reported similar findings to those of Çiftçi et al. [36] (*P* = 0.42). Both block applications in these studies were carried out under USG guidance with 30 mL 0.25% bupivacaine. The principal differences between these two studies and the present research lie in the volume of local anesthetic used and that the RIB was compared with different regional techniques. Different volumes and concentrations of local anesthetic are used in the RIB, and there are still no definite data concerning the optimal dose selection. In their study of the RIB optimal dose, Denk et al. [35] compared various ropivacaine concentrations. Those authors reported no superiority over one another of 0.2%, 0.3%, and 0.4% concentrations. Bupivacaine, which is available in Türkiye, was used for the ESPB and RIB in the present study. However, there are no optimal dose studies for bupivacaine in RIB applications. Inconsistent findings have been reported in ESPB-related dose and concentration studies. Demirel et al. [36]. compared 0.375% bupivacaine at differing volumes (30 mL and 20 mL) in breast surgery, and reported similar intraoperative and postoperative 24-hour opioid consumptions and postoperative NRS values. Altıparmak et al. [37] compared the efficacy of ESPB with 20 mL 0.375% bupivacaine and 0.25% in breast surgery and reported similar intraoperative fentanyl consumptions. However, postoperative tramadol consumption and NRS scores were lower in the 0.375% bupivacaine group (*P* < 0.001). Although 20 ml 0.375% bupivacaine was more effective in the postoperative period than 0.25% in that study, Park et al. [38] reported similar postoperative opioid consumptions and NRS scores between the same concentrations. In agreement with the previous literature, we elected to employ the lowest volume and concentration of local anesthetic capable of providing the most effective analgesia in both block techniques. We therefore used 20 mL 0.25% bupivacaine for both ESPB and RIB. However, further studies investigating the optimal dose and concentration are still needed.

Publications concerning RIB applications in breast surgery are scarce (Table 6). To the best of our knowledge, the only prospective, randomized, controlled study comparing the postoperative analgesic efficacy of the RIB and ESPB in breast surgery is Deng et al.’s [4] study from 2021. That research involved 90 patients who underwent ESPB, RIB, and deep serratus anterior plane block (SAPB). The authors employed 20 mL 0.5% ropivacaine under USG guidance for all the blocks. The SAPB was performed with the patient in the supine position, while ESPB was performed from the T4 level and the RIB from the T5-6 level with the patients in the lateral decubitus position. We also applied the ESPB from the T4 level and the RIB from the T5-T6 level in the present study. That was because studies have shown sufficient analgesic efficacy in ESPB applications at the T4 level in breast surgery [18, 21]. In contrast to that study, however, we applied both blocks with the patients in the prone position. We think that needle stabilization is more comfortable in block applications with the in-plane technique, and that the prone position in sedated patients is preferable in terms of patient comfort to the lateral decubitus position. Total 24-hour tramadol consumptions in that research were significantly lower in the groups undergoing ESPB (273.67 ± 36.90 mg) and RIB (269.67 ± 48.75 mg), than in the patients undergoing SAPB (314.33 ± 18.88 mg) (*P* < 0.001). NRS values were also lower in the patients who underwent ESPB and RIB than in those who underwent SAPB. However, similarly to the present study, comparison of the ESPB and RIB groups revealed no significant difference in terms of postoperative opioid consumptions or NRS values. In contrast to the present study, Deng et al. [4] employed ropivacaine as a local anesthetic, and this variation may depend on clinicians and countries’ routine procedures.

**Table 6:** Summary of studies investigating RIB and other regional blocks for postoperative analgesia in breast surgery

Author	Patient Group	Surgery Type	Block Level	Local Anesthetic Used	Postoperative Analgesia Management	Outcome
Altıparmak et al. 2020 [30]	Total: 56 patients Control (*n* = 28) RIB (*n* = 28)	MRM	RIB; T5	30 mL of 0.25% Bupivacaine	Morphine PCA	Postoperative opioid consumption was found to be lower in the RIB group, whereas NRS scores were similar between the two groups.
Çiftçi et al. 2021 [33]	Total: 90 patients Control (*n* = 30) RIB (*n* = 30) PSPB (*n* = 30)	BCS	RIB; T5-T6	30 mL of 0.25% Bupivacaine	Fentanyl PCA	RIB and PSPB demonstrated comparable efficacy in postoperative analgesia.
Denk et al. 2021 [4]	Total: 90 patients ESPB (*n* = 30) RIB (*n* = 30) SAPB (*n* = 30))	MRM	RIB; T5-T6 ESPB; T4 SAPB; T4-T5	20 mL of 0.5% Ropivacaine	Tramadol	ESPB and RIB showed superior postoperative analgesic efficacy compared to SAPB. However, RIB and ESPB were comparable in terms of postoperative opioid consumption and NRS scores.
Kültüroğlu et al. 2024 [34]	Total: 72 patients LA infusion (*n* = 24) RIB (*n* = 24) IPPB+PSPB (*n* = 24)	MRM	RIB; T5-T6	30 mL of 0.25% Bupivacaine	Tramadol PCA	The lowest total tramadol consumption and NRS scores were observed in the RIB group. However, RIB and IPPB+PSPB groups were similar in terms of total tramadol consumption.

*MRM *Modified radical mastectomy, *BCS * Breast-conserving surgery, *PCA * Patient-controlled analgesia, *NRS * Numeric Rating Scale, *RIB * Rhomboid intercostal block, *PSPB * Pectoserratus block, *ESPB* Erector spinae plane block, *SAPB* Serratus anterior plane block, *IPPB* Interpectoral–pectoserratus block, *LA* Local anesthetic

No block-related complications were observed in this study. We attribute this to the blocks being performed by experienced practitioners and under USG guidance.

When interpreting the results of this study, it should be noted that we employed a multimodal analgesic regimen for our patients. This could be potential limitation of our study. In the context of robust multimodal analgesia, ESPB and RIB provide similar postoperative opioid consumption and pain scores, and detecting finer differences between techniques might require either a different design (e.g., less intense systemic analgesia) or a larger sample size.

Given the very low absolute opioid requirements observed in this study, even small numerical differences in morphine consumption may appear relatively large in percentage terms. Large meta-analytic and anchor-based studies have suggested minimal clinically important differences of approximately 5–10 mg intravenous morphine equivalents across a wide range of surgical populations [39]. We therefore deliberately chose a very conservative non-inferiority margin of 0.545 mg, corresponding to roughly 15–20% of the expected mean 24-h morphine consumption in this population, as the maximum tolerable loss of efficacy that we would still consider clinically acceptable in exchange for the potential practical advantages of RIB over ESPB.

In addition to the limitation discussed above, several other limitations should be acknowledged. These include the fact that no dermatomal examination was performed in order to evaluate efficacy following the block applications. Although determining block effectiveness through dermatomal examination in fascial plane blocks is not absolutely essential, it might have been useful for assessing block success. However, we did not consider dermatomal examination appropriate since the patients undergoing breast surgery were largely in the fragile group receiving cancer treatment.

Although there was no difference in the number of patients with or without axillary dissection between the groups in our study, the surgical heterogeneity of the patients is one of the limitations of the study.

Another limitation is that although our primary objective was to evaluate acute pain, we employed scales measuring chronic pain and quality of life in the postoperative period [40, 41].

## Conclusion

Within a standardized multimodal analgesia regimen for unilateral breast surgery, ESPB and RIB yielded comparable postoperative opioid consumption and pain scores. Clinicians may therefore select either technique according to their own expertise and institutional practice.

## Data Availability

The datasets used and/or analyzed during the current study are available from the corresponding author on reasonable request.
